# Reliable and rapid characterization of functional *FCN2* gene variants reveals diverse geographical patterns

**DOI:** 10.1186/1471-2350-13-37

**Published:** 2012-05-17

**Authors:** Olusola Ojurongbe, Eman Abou Ouf, Hoang Van Tong, Nguyen L Toan, Le H Song, Paola R Luz, Iara JT Messias-Reason, Dennis Nurjadi, Philipp Zanger, Jürgen FJ Kun, Peter G Kremsner, Thirumalaisamy P Velavan

**Affiliations:** 1 Institute of Tropical Medicine, University of Tübingen, Tübingen, Germany; 2 Ladoke Akintola University of Technology, Ogbomoso, Nigeria; 3 Department of Pathophysiology, Vietnam Military Medical University, Hanoi, Vietnam; 4 Tran Hung Dao Hospital, Hanoi, Vietnam; 5 Laboratório de Imunopatologia Molecular-Hospital de Clínicas, Federal University of Paraná, Curitiba, Brazil

**Keywords:** FRET, Ficolin-2, Genotypes, Haplotypes, Distribution

## Abstract

**Background:**

Ficolin-2 coded by *FCN2* gene is a soluble serum protein and an innate immune recognition element of the complement system. *FCN2* gene polymorphisms reveal distinct geographical patterns and are documented to alter serum ficolin levels and modulate disease susceptibility.

**Methods:**

We employed a real-time PCR based on Fluorescence Resonance Energy Transfer (FRET) method to genotype four functional SNPs including *-986 G > A* (#rs3124952), *-602 G > A* (#rs3124953), *-4A > G* (#rs17514136) and *+6424 G > T* (#rs7851696) in the ficolin-2 (*FCN2)* gene. We characterized the *FCN2* variants in individuals representing Brazilian (n = 176), Nigerian (n = 180), Vietnamese (n = 172) and European Caucasian ethnicity (n = 165).

**Results:**

We observed that the genotype distribution of three functional SNP variants (−*986 G > A, -602 G > A* and *-4A > G*) differ significantly between the populations investigated (*p* < 0.0001). The SNP variants were highly linked to each other and revealed significant population patterns. Also the distribution of haplotypes revealed distinct geographical patterns (*p* < 0.0001).

**Conclusions:**

The observed distribution of the *FCN2* functional SNP variants may likely contribute to altered serum ficolin levels and this may depend on the different disease settings in world populations. To conclude, the use of FRET based real-time PCR especially for *FCN2* gene will benefit a larger scientific community who extensively depend on rapid, reliable method for *FCN2* genotyping.

## Background

Innate immune system has a pivotal role as a first line defense against any invading pathogens without previous exposure while signaling towards a specific adaptive immune response [1]. Ficolin-2 encoded by the *FCN2* gene located on chromosome 9q34 has eight exons and represent one such vital innate immune recognition protein that are involved in immune defense against pathogens [2]. The exon 1 of the *FCN2* encodes a smaller segment of the N-terminal amino acids whereas exons 2 and 3 encode the collagen-like domain. The fourth exon encodes the linker region and exons 5–8 encode the fibrinogen like binding domain (FBG). A part of exon 8 encodes the fibrinogen like binding domain and the rest encodes the 3' UTR region and the SNPs at exon 8 were believed to cause specific alterations of the FBG domain thus affecting the binding to the ligands [3]. The ficolin-2 has a fibrinogen like domain that interacts with sugar moieties such as N-acetylglucosamine (GIcNAc) and other acetylated compounds present on the surface of the pathogens leading to complement activation [3,4]. Also ficolin-2 is known to bind specifically to lipotechoic acid on the cell wall of gram positive bacteria and to 1, 3-β-D-glucan, components of fungal and yeast cell walls [5,6]. Similar to Mannose binding lectin (MBL), ficolins are capable of binding to pathogen-associated molecular patterns (PAMPs) and in association with the enzymes MBL-associated serine proteases (MASP1 and MASP2), ficolin-2 initiates the complement lectin cascade [7]. Studies have documented that three promoter polymorphisms of the *FCN2* gene (−*986 G > A, -602 G > A, and -4A > G*) are significantly correlated in a gene dose dependent manner with either a twofold decrease or increase in the ficolin-2 concentrations in the serum [8]. Also the *FCN2* variant in exon 8 (*+6424 G > T*) with amino acid substitution (Ala258Ser) has been demonstrated earlier to have elevated binding capacity to GIcNAC than the major allele [4,9].

The SNPs in the *FCN2* gene had been studied in different world populations [10] and it was demonstrated that single nucleotide polymorphisms at positions–*986 G > A, –602 G > A* and *–4A > G* in the promoter region and SNP at position *+6424 G > T* in the coding region were significantly associated to several infectious diseases such as respiratory infections in children, rheumatic fever, rheumatic heart disease, leprosy, malaria [11-15], hepatitis B [16] and in cutaneous leishmaniasis [17]. Because of its vital contribution in host immune defense, *FCN2* genotyping provides genetic clues to elucidate the association with different infectious and autoimmune diseases. In recent decades the hybridization-based methodology using real-time PCR is widely used for SNP detection. Various probes such as hybridization probe (Hybprobe), SimpleProbe and molecular beacons [18,19] are utilized for real-time PCR based genotyping approaches. In the present study, we genotyped and characterized four *FCN2* functional SNP variants (−*986 G > A, –602 G > A, –4A > G* and *+6424 G > T*) in West Africans from Nigeria, South Americans from Brazil, South East Asians from Vietnam and Caucasians from Germany using an available rapid, reliable and cost effective FRET-RT-PCR methodology.

## Methods

### Genomic DNA isolation

DNA samples utilized for this study represented cohort from our previous study for Vietnamese population [16] and from our unpublished data for Nigerian, Brazilian and Caucasian individuals. The characterization of the *FCN2* functional variants were investigated in individuals representing Brazilian (n = 176), Nigerian Youruba individuals (n = 180), Vietnamese (n = 172) representing Viet ethnicity and European Caucasian ethnicity (n = 165). Genomic DNA was isolated using QIAamp DNA -Blood Mini kit (Qiagen, Hilden, Germany). Informed written consent was obtained from the study volunteers. The study was approved by the ethics committee of the Hospital de Clínicas in Curitiba, Brazil, local ethics committee of the Ministry of Health, Abeokuta Ogun State, Nigeria, the institutional review board of the Tran Hung Dao Hospital, Hanoi, Vietnam, and the ethics committee of the Medical Faculty in Tübingen, Germany.

### *FCN2* genotyping

Three SNPs – *986  G> A* (rs3124952),–*602 G > A* (rs3124953) and – *4A > G* (rs17514136) in the promoter region and one SNP in exon 8 +*6424 G > T* (rs7851696) were genotyped using real-time polymerase chain reaction (real-time PCR) based on the allelic discrimination principle utilizing florescence resonance energy transfer (FRET) principle. In FRET, both anchor and sensor probes were labeled with either fluorescein or cyanine dye as applicable to the orientation of the SNP detection. The region of interest is amplified by forward and reverse primer pairs. The SNP specific sensor probes were designed one nucleotide apart from the anchor probe to facilitate the energy transfer between the two fluorescent dyes in proximity. Both probes were designed to be localized on the same DNA strand to anneal on the target sequence in a head-to tail arrangement. During the melting phase, energy transfer referred to as FRET occurs. This excitation energy is transferred from the anchor to the sensor probe and the emitted fluorescence is detected at 660 hp wavelength during the melting phase. Gradual increase in temperature decreases the fluorescence intensity as one of the probes melt off leaving the two fluorescent dyes apart. The sensor probe with a clear match can still anneal to the target SNP but it melts off at a higher temperature contrary to the mismatch that melts off at a lower melting temperature. Therefore the difference in the melting temperature remains as a basis to differentiate the genotypes. All PCR reactions followed by melting curve analysis were performed using the Rotor Gene ver.6.1.81 software (Corbett, Sydney, Australia). PCR amplifications were carried out in a 20 μl volume with 50 ng of DNA, 1X QuantiTech multiplex PCR NoRox Master Mix (Qiagen, Hilden, Germany) with SNP specific primer pairs and SNP specific probes in a defined concentration (Table 1). Thermal cycling parameters were: initial denaturation and Taq polymerase activation at 95°C for 15 min, followed by 40 cycles of two-step cycling with denaturation at 94°C for 1 min, annealing and extension at 60°C for 1 min. After subsequent amplification, melting temperature analysis was performed. The cycling parameters followed were: denaturation at 95°C for 1 minute and immediate cooling to 40°C for 1 minute. This step is followed by increasing each degree with a hold time of 2 sec/degree until 90°C and a final cooling at 40°C for 1 minute. A negative and positive control was integrated for each run. All the reactions including the positive and negative controls were analyzed using the Rotor Gene 3000 (Corbett, Sydney, Australia) and the genotypes were validated by analyzing the melting temperature of the probes with the Rotor-Gene 6.1.81 Software version (Corbett, Sydney, Australia). The primer pairs and hybridization probes along with the allele specific annealing are summarized in Table 1.

**Table 1:** **Primers and Probes and observed melting temperatures employed for* FCN2 *SNP detection**

**Position**	**Primer/probe (Conc.)**	**Oligonucleotide sequence (5′- 3′)**	**Genotypes (Tm)**
−986 *G* > *A* (rs3124952)	Forward (0.5 μM)	5′-GGGTCACAGTTTAAAATCCTTCTACT-3′	
	Reverse (0.05 μM)	5′-CGTATACCTAAAGCCCCCAGA-3′	GG (73°C)
	Sensor (0.25 μM)	CY5-GCCACCTGC**[C]**GCCATCG-PH	GA (73°C/64°C)
	Anchor (0.25 μM)	5′CCTCCCACTACCACCACCGCACCC-FL	AA (64°C)
−602 *G* > *A* (rs3124953)	Forward (0.05 μM)	5′-CAAGGTCTCCCCTTCAGATG-3′	
	Reverse (0.5 μM)	5′-CATGAGCAGACTTGGGACT-3′	GG (60°C)
	Sensor (0.15 μM)	5′-CCTCCTGTTC**[A]**TGTGCCCC-FL 3′	GA (60°C/67°C)
	Anchor (0.15 μM)	CY5-GTGCTCTACATACTGCCCCAGGAAACAG-PH	AA (67°C)
−4 *A* > *G* (rs17514136)	Forward (0.125 μM)	5′-GGAAGCGGCTGTCACTC-3′	
	Reverse (0.5 μM)	5′-CCCTTACCTGGACAGGTGT-3′	AA (64°C)
	Sensor (0.15 μM)	5′AGCAAAGACCAGA**[A]**GAGATGGA-FL	AG (64°C/58°C)
	Anchor (0.15 μM)	CY5-CTGGACAGAGCTGTGGGGGTC-PH	GG (58°C)
+6424 *G* > *T* (rs7851696)	Forward (0.5 μM)	5′-TGCCTGTAACGATGCTCA-3′	
	Reverse (0.05 μM)	5′-TGTATCCTTTCCCCGACTT-3′	GG (65°C)
	Sensor (0.15 μM)	5′-GAAACATCACAG**[C]**ACAATTTCC-FL	GT (65°C/55°C)
	Anchor (0.15 μM)	CY5-GTGTTAAGATCATTGTCCTGGTCTTTGGT-PH	TT (55°C)

*FL* fluroscein, *CY5* cyanin, *PH* phosphorylated end.

In addition, the observed genotypes of *FCN2* by real- time PCR were reconfirmed for their SNP variant by direct sequencing of the respective DNA fragments. Thirty randomly chosen DNA samples each from Brazilian, Nigerian, Vietnamese and Caucasians individuals were PCR amplified with appropriate primer pairs. The PCR products were purified using ExoSAP-IT® (Affymetrix, Inc Ohio, USA) and 1 μl of the purified product were directly used as templates for sequencing, using the BigDye terminator v. 2.0 cycle sequencing kit (Applied Biosystems, USA) on an ABI 3100 DNA sequencer, according to the manufacturer’s instructions. DNA polymorphisms were identified when assembled with the reference sequence of *FCN2* gene (NG_011649.1) using the BioEdit http://www.mbio.ncsu.edu/BioEdit/bioedit.html] program.

### Statistical analysis

Data had been analysed by StatView (http://www.statview.com) and the level of significance was set to *P* < 0.05. Normal Chi square and two tailed Fisher’s exact tests were executed to determine the differences in genotype and haplotype distributions in different ethnicities. Genotype or haplotype frequencies were determined by simple gene counting and by using the expectation-maximum (EM) algorithm. The significance of deviations from Hardy-Weinberg equilibrium was tested using the random-permutation procedure as implemented in the Arlequin v. 3.5.1.2 software. (http://lgb.unige.ch/arlequin). Linkage disequilibrium (LD) analysis was performed using Haploview v. 3.2 program.

## Results

**Figure 1:**
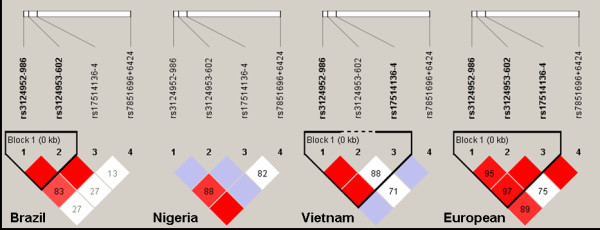
**Haploview plot illustrating the linkage disequilibrium of the*FCN2*gene variants in different ethnicities.** At the top, the SNPs are shown according to their succession from the start of the translation of the *FCN2* gene. Open squares indicate a high degree of LD (LD coefficient D’ = 1) between pairs of markers. Numbers indicate the D’ value expressed as a percentile. Red squares indicate pairs in strong LD with LOD scores LD ≥ 34; pink squares, D’ = 1 with LOD ≤ 2; white squares, D’ < 1 with LOD ≤ 1.

**Figure 2:**
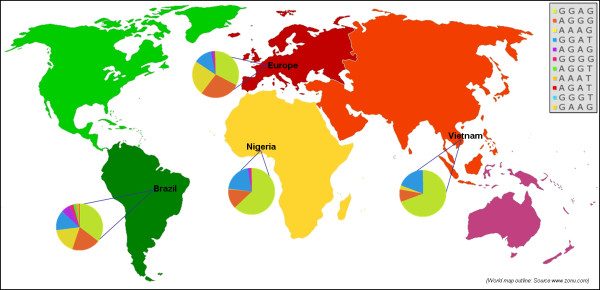
**Distribution of*FCN2*haplotypes in world populations.**

The observed genotypes were reproducible for their SNP status by direct sequencing. The primer pairs and hybridization probes employed for respective *FCN2* allele (−*986 G > A, -602 G > A, -4A > G* and *+6424 G > T*) detection are summarized in Table 1. The geographical distribution of *FCN2* genotypes in four different ethnicities is summarized in Table 2. The SNP variants–*986 G > A, –602 G > A* and –*4A > G* differed significantly in the populations tested (*p* < 0.0001) Table 2. The *–602 G > A* SNP variant was not in Hardy Weinberg equilibrium in samples representing Nigerian and Vietnamese ethnicity. A similar distribution of genotypes was observed for the *FCN2* variant *+6424 G > T* irrespective of ethnicity. Linkage disequilibrium analysis revealed strong allelic combinations at positions for –*986 G > A* and *–602 G > A* in Brazilian population; whereas the –*986 G > A* and *–4A > G* revealed strong allelic combinations in both Nigerian and Vietnamese population. In European Caucasian population, all three SNP variants–*986 G > A* (#rs3124952), –*602 G > A* (#rs3124953), –*4A > G* (#rs17514136)) in the promoter region revealed strong allelic combinations Figure 1. The geographical distribution of *FCN2* haplotypes in four different ethnicities is summarized in Table 3 and Figure 2.

**Table 2:** **Observed genotypes in four* FCN2 *gene variants in different world populations**

**SNP variant**		**Brazilian**		**Nigerian**		**Vietnamese**		**Europeans**		
		**n =176**	Frequency*	**n =180**	Frequency*	**n =172**	Frequency*	**n =165**	Frequency*	***P*Value**
-986 G/A	GG	47	0.27	128	0.71	135	0.77	43	0.26	<0.0001
	GA	90	0.51	49	0.27	36	0.20	69	0.42	
	AA	39	0.22	3	0.02	1	0.01	53	0.32	
-602 G/A	GG	116	0.66	179	0.99	162	0.92	95	0.58	<0.0001
	GA	51	0.29	1	0.01	10	0.06	64	0.39	
	AA	9	0.05	0	0	0	0.00	6	0.03	
-4A/G	AA	103	0.59	130	0.72	144	0.82	87	0.53	<0.0001
	AG	58	0.33	46	0.26	28	0.16	61	0.37	
	GG	15	0.08	4	0.02	0	0.00	17	0.10	
+6424 G/T	GG	116	0.66	116	0.64	111	0.63	126	0.76	NS
	GT	55	0.31	53	0.29	53	0.30	32	0.19	
	TT	5	0.03	11	0.07	8	0.05	7	0.05	

* Frequencies may not add up to 1.00 due to rounding errors.

**Table 3:** **Observed haplotypes of*FCN2*gene variants in different world populations**

**Haplotype**	**Brazilian**		**Nigerian**		**Vietnamese**		**Europeans**		
	**n =352**	Frequency*	**n =360**	Frequency*	**n =344**	Frequency*	**n =330**	Frequency*	***P*Value**
**G G A G**	125	0.355	226	0.628	237	0.689	108	0.327	<0.0001
**A G G G**	69	0.196	49	0.136	28	0.081	93	0.282	<0.0001
**A A A G**	62	0.176	1	0.003	10	0.003	74	0.224	<0.0001
**G G A T**	49	0.139	74	0.205	69	0.200	44	0.133	0.012
**A G A G**	21	0.059	5	0.013	0	0.000	6	0.019	<0.0001
**G G G G**	10	0.028	4	0.011	0	0.000	2	0.006	0.003
**A G G T**	9	0.025	0	0.000	0	0.000	0	0.000	<0.0001
**A A A T**	7	0.019	0	0.000	0	0.000	1	0.003	0.001
**A G A T**	0	0.000	0	0.000	0	0.000	1	0.003	NS
**G G G T**	0	0.000	1	0.003	0	0.000	0	0.000	NS
**G A A G**	0	0.000	0	0.000	0	0.000	1	0.003	NS

* Frequencies may not add up to 1.00 due to rounding errors.

## Discussion

Ficolins are able to bind to specific ligand such as pathogen-associated molecular patterns (PAMPs) expressed on the surface of pathogens and trigger the complement cascade by interacting with mannose binding lectin associated serine proteases (MASPs) to swiftly contain the early infection. This study based on FRET- realtime PCR, characterized distinct genetic geographical patterns in the four functional *FCN2* gene variants that are documented to alter the serum ficolin levels and disease susceptibility. In recent years, many studies have used larger cohort to investigate the contribution of *FCN2* SNPs (−*986 G > A, –602 G > A, –4A > G* and *+6424 G > T*) to diseases outcome [11-15]. All these studies had employed the standard sequencing analysis and Taqman approach for investigation of these vital *FCN2* SNPs. With increasing sample size, the mechanism of allelic discrimination and detection methodologies remain laborious and time consuming. In comparison to direct sequencing, our approach augments the attributes of rapid and reliable characterization of *FCN2* variants. Moreover the employed methodology utilizes simple data analysis for accurate genotype calling with a limited turn over time of three hours for 72 samples for one single run. Of significance is that genotypes observed using the FRET-RT PCR method were reconfirmed for their polymorphism status by direct sequencing that justifies the reliability of this study procedure. In addition, the observed genotype distributions for the West African Nigerian (n = 180) and European Caucasian ethnicities (n = 165) across all four functional *FCN2* variants (−*986 G > A, –602 G > A, –4A > G* and *+6424 G > T*) were consistent with published variant frequencies in West African Ghana population (n = 50) and European Denmark individuals (n = 60) [10]. These consistent results yet auguments the methodology as well as the genetic charcterization utilized in the study procedure. A previous study had reported the use of Taqman probes for *FCN2* gene polymorphisms detection with low conversion rate and difficulty in their assay optimization [8,13]. The difference between the melting peaks of the major and minor alleles in our study were between 6°C to 10°C which makes it obviously possible to distinguish between the homozygotes and the heterozygote.

Earlier studies have documented that ficolin-2 serum concentration was distributed in a gene dose-dependent manner, i.e. homozygotes had either the highest or the lowest ficolin-2 concentrations, whereas heterozygotes had intermediate concentrations [9]. Three polymorphism in the promoter region of the *FCN2* gene (−*986 G > A, –602 G > A,* and *–4A > G*) were associated with variation in ficolin-2 serum levels, where as the *FCN2* variant in exon 8 (*+6424 G > T*) with amino acid substitution non-polar to polar (Ala258Ser) has been demonstrated to have elevated binding capacity to GIcNAC than the major allele [4,9]. From our results, we observed that only the genotypes of the promoter region (−*986 G > A, –602 G > A,* and *–4A > G*) of the *FCN2* gene is distributed significantly in the different world populations and all these variants are reported to be associated with varying serum ficolin-2 levels. In line with the documented studies, our results reveal a homozygous excess in the investigated Nigerian and Vietnamese populations. The *+6424 T* allele that was identified to have an elevated binding capacity to GIcNAC was detected in low frequencies irrespective of the studied ethnicities. We also observed that –*602 G > A* genotypes were mostly homozygous (−*602 GG*) in Nigerian and Vietnamese populations and were not in hardy Weinberg equilibrium. This may aid the researchers to disregard this functional *FCN2* variant (−*602 G > A*) for investigation in any case–control studies in Nigerian and Vietnamese populations. From our recent study in a Nigerian cohort, we observed that only the heterozygous variants –*986 G > A,* and *–4A > G* in the promoter region contributed to increased susceptibility to schistosomiasis (our unpublished data) whereas none of these variants were observed to contribute to hepatitis B disease outcome in a gene dose dependent manner [16].

The haplotypes distribution differed significantly across continents. The observed frequency of *FCN2* haplotypes in European and Brazilian populations were far higher in comparison to haplotypes representing Vietnamese and Nigerian ethnicity. Moreover the haplotype patterns remained similar in Europeans and Brazilians. The recent genetic admixture by the inflow of European and other ethnic descendents to Brazil may be a plausible explanation for the observed trend. The frequency of haplotypes remained far less in Nigeria and Vietnam compared to other investigated populations and this may be due to a reduced ethnic admixture in these populations. Our earlier studies have inferred that the reconstructed haplotypes (−*986/-602/-4/+6424*) were shown to influence the predisposition to HBV outcome, leprosy and in rheumatoid heart disease [12,14,16] and in cutaneous leishmaniasis [17]. In all these documented studies, only the haplotypes were observed to significantly contribute to the clinical manifestation of the diseases. All this direct towards a contribution of haplotypes to disease association may possibly be of far more significance rather than a genotype dependent association. Also it is likely that clinical significance of these haplotypes may vary also depending on disease context.

## Conclusion

In summary, this study on the use of FRET based real-time PCR especially for *FCN2* gene will benefit a larger scientific community who extensively depend on rapid, reliable method for *FCN2* genotyping. To conclude, the observed ethnic differences in the *FCN2* functional SNP variants is believed to affect the concentration and the function of the ficolins, that may probably contribute to the pathophysiological significance in different disease settings.

## Competing interests

The authors declare no conflict of interest.

## Authors’ contributions

OO established the RT-PCR FRET methodology and involved in data analysis; EOA established and standardized the methodology and genotyped Nigerian population; HVT genotyped Vietnamese samples; NLT collected Vietnamese samples and involved in the study design; LHS collected Vietnamese samples and involved in study design; PRL genotyped Brazilian samples; IJTMR collected Brazilian samples and involved in study design; DN genotyped Caucasisan samples; PZ collected Caucasian samples and involved in study design; JFK involved in experimental design and data analysis; PGK experimental design and edited the manuscript; VTP initial ideas, experimental design, data analysis and drafted the manuscript. All authors read and approved the final manuscript.

## Pre-publication history

The pre-publication history for this paper can be accessed here:

http://www.biomedcentral.com/1471-2350/13/37/prepub
